# Task-Contingent Persistence is Related to Better Performance-Based Measures in Patients with Chronic Musculoskeletal Pain

**DOI:** 10.1155/2020/1765456

**Published:** 2020-06-16

**Authors:** Cyrille Burrus, Philippe Vuistiner, Bertrand Léger, Gilles Rivier, Roger Hilfiker, François Luthi

**Affiliations:** ^1^Department for Musculoskeletal Rehabilitation, Clinique Romande de Réadaptation Suva, Avenue Grand-Champsec 90, 1950 Sion, Switzerland; ^2^Institute for Research in Rehabilitation, Clinique Romande de Réadaptation Suva, Avenue Grand-Champsec 90, 1950 Sion, Switzerland; ^3^School of Health Science, University of Applied Sciences and Arts Western Switzerland Valais (HES-SO Valais-Wallis), 1950 Sion, Switzerland; ^4^Department of Physical Medicine and Rehabilitation, Orthopaedic Hospital, Lausanne University Hospital, Rue du Bugnon 21, 1011 Lausanne, Switzerland

## Abstract

**Purpose:**

Pacing, avoidance, and overdoing are considered the three main behavioral strategies, also labeled activity patterns. Their relationship with functioning of patients with chronic pain is debated. The purpose of this study was to measure the influence of activity patterns on lifting tasks commonly used in daily life.

**Method:**

We performed a monocentric observational study and included patients performing Functional Capacity Evaluation (FCE). Avoidance, pacing, and persistence were assessed with using the Patterns of Activity Measures–Pain (POAM-P). Maximal safe performance was measured for floor-to-waist, waist-to-overhead, horizontal lift, and carrying with dominant-hand tests according to the FCE guidelines. Descriptive statistics, associations of POAM-P subscales with various sociodemographic variables, and correlations are presented. Standard multiple linear regression models were applied to measure the associations between FCE tests and POAM-P subscales, adjusting for the following potential confounders: age, gender, body mass index (BMI), pain severity, trauma severity, localization of injury, and education.

**Results:**

Persistence was significantly positively associated with performance on the 4 FCE tests: floor-to-waist (coefficient = 0.20; *p*=0.001), waist-to-overhead (coefficient = 0.13; *p*=0.004), horizontal lift (coefficient = 0.31; *p* ≤ 0.001), and dominant-handed lifting (coefficient = 0.19; *p*=0.001). Pacing was found to have a negative influence on the carrying dominant-hand test (coefficient = –0.14; *p*=0.034), and avoidance was not found to have an influence on the 4 FCE tests.

**Conclusion:**

This study shows that task-persistence pattern is positively associated with physical performance in FCE, whereas pacing can have a negative influence on some tests.

## 1. Introduction

The influence of behavior on the development and perpetuation of chronic pain has been the subject of debate for many years [1, 2]. There are generally 3 main behavioral strategies considered [3] also labeled *activity patterns*, which can be used to varying degrees and combinations by patients with chronic pain, depending on the context [4]: avoidance (defined as a decrease or escape of physical activity due to fear of movement or fear of pain), pacing (described as a regulation using alternating periods of activity and rest often with the objective of controlling pain or fatigue), and persistence (described as continuing activity despite pain with the risk of detrimental effects). Persistence, in contrast to other patterns, has been given various designations: *endurance*, *overactivity*, *persistence*, *confrontation*, or *overdoing* in the original Cane study [5]. The construct of the overdoing pattern measured with the Patterns of Activity Measure– Pain (POAM-P) used in this study has been shown by Kindermans to reflect a task-contingent persistence pattern [6]. To be in agreement with both Cane's choice and the underlying construct, this pattern will be labeled *overdoing/persistence* in this study. The relationship between these patterns and the functioning of patients with chronic pain remains subject to significant controversy [7–9]. One of the reasons for this may be the discrepancies often observed between subjective measures of functioning and observational measures [10, 11]. For instance, disability questionnaires are consistently associated with the intensity and duration of pain, while observational measures of daily living activities often show little or no difference [6, 12]. Improving physical capacities is an important therapeutic objective of rehabilitation. In particular, knowledge of one's own ability to carry a load safely has multiple implications for daily life and social participation of people with chronic pain: for example, shopping, carrying a child, or performing different physical tasks in many jobs. In clinical conditions, it is sometimes challenging to transfer standardized and controlled measurement conditions that are used in the laboratory [13]. Consequently, the level of maximal safe physical performance achieved by patients with chronic pain may be particularly hard to determine [14]. Functional Capacity Evaluation (FCE), frequently employed in rehabilitation to determine physical performance, is one of the ways to overcome this discrepancy [15]. It is based on standardized measurements, with a clear definition of a safe maximal performance, and has been validated [15]. It has good to excellent reliability [16–18]. Self-efficacy and various biological factors such as age, body mass index (BMI), and gender are known to influence FCE performance [15, 19–21]. The relationship between psychological variables and FCE is debated [15, 21]. To date, no studies have evaluated the possible associations between behavioral activity patterns and FCE in patients with chronic musculoskeletal pain. In a previous study, however, we found that the overdoing/persistence pattern was positively associated with commonly used performance-based measures (walking, lifting (pile-test), and physical fitness). No consistent association was found with the avoidance pattern, and pacing was negatively associated with walking performance [22]. In another setting, pacing activity pattern was associated with lower levels of perceived disability [3]. There is a need to better understand behavioral factors influencing physical activity and the barriers that could explain the lack of improvement of physical performances, despite the best-designed rehabilitation program [23].

The aim of our study was to investigate the influence of activity patterns on patients with chronic musculoskeletal pain during FCE by using the POAM-P questionnaire and to answer the following question. What are the associations between the 3 activity patterns and the FCE lifting performances in patients with chronic musculoskeletal pain? Based on previous research addressing observational measurements [22], our hypothesis was that overdoing/persistence would be positively associated with FCE, whereas pacing and avoidance would not show consistent associations with FCE.

## 2. Materials and Methods

### 2.1. Study Design

We conducted an observational study at the Clinique Romande de Réadaptation, a rehabilitation center in the French-speaking part of Switzerland.

### 2.2. Participants

Any patient of working age (18–65 years old) who had an FCE from October 2013 to April 2018 was eligible. As previously described [15], exclusion criteria were patients with spinal cord or traumatic brain injuries, incapable of judgment or under legal custody, and with inability to complete the questionnaire in French. The setting of rehabilitation [15, 22] of patients consisted of patients from French-speaking urban or more rural regions of Switzerland are referred from by general practitioners, surgeons, or insurance medical advisors when they present with chronic musculoskeletal pain (≥3 months), functional impairments, and inability to return to work after orthopedic injuries following work, traffic, sport, or leisure activities [15, 22]. Patients undergo outpatient rehabilitation close to their residence and are sent to a specialized rehabilitation center in case of unfavorable evolution or they are unable to resume their professional activity. The aim of the therapeutic program is to manage patients using a multidisciplinary biopsychosocial approach according to the practice recommendations for chronic pain patients [24]. Individual and group sessions are offered including physical and occupational therapies. Therapies are based on graded and functional trainings, with trainings in professional training workshops, social advice, and psychological components with an average of 4 psychological cognitive-behavioral therapy periods. The mean length of stay is between four and five weeks, with around four hours of therapies every working day. The inclusion period ran from October 2013 to April 2018. If a patient was treated twice during this period, we considered data from only the first rehabilitation.

All participants read and signed an informed consent form to allow access and the use of their clinical data for the present study. The protocol was approved by the ethical committee of the local medical association (Commission Cantonale Valaisanne d'Ethique Médicale, CCVEM 034/12), and the study was performed in accordance with the ethical standards listed in the 2008 Declaration of Helsinki.

### 2.3. Participants' Data

Patients' sociodemographic data and variables of interest were chosen for their possible confounding influence on the FCE performances [21]. They were collected at the time of admission by the doctor in charge and included (1) age, (2) gender, (3) body mass index (BMI), (4) injury location (upper limb, lower limb, spinal, or multiple-site injuries), (5) trauma severity based on the Abbreviated Injury Scale score (minor injury versus moderate or serious injury) [25], (6) duration from injury to rehabilitation (days), (7) first language (French versus others), (8) education level (≥9 years of schooling: yes versus no), (9) employment contract (yes versus no), and (10) work-related injury (yes versus no).

### 2.4. Questionnaires

The Hospital Anxiety and Depression Scale (HADS) [26] was used to assess depression and anxiety symptoms. The HADS consists of 2 subscales, each containing 7 questions (range, 0–21), with a higher score suggesting higher depressive or anxious symptoms [27]. We used the validated French version [28].

For pain assessment, we used 2 subscales of the Brief Pain Inventory (BPI) questionnaire. The severity subscale measured pain at its “worst,” “least,” “average,” and “now” state with 4 numeric scales and was scored as the mean (scale, 0–10), with a high score indicating high severity [29–31]. The interference subscale measured how much pain interferes with seven daily activities. It was scored as the mean of the seven interference items (scale, 0–10), with a high score indicating a high pain-related disability [29–31].

Perceived functional ability was measured by the Hand Function Sort (HFS) and the Spinal Function Sort (SFS), which are pictorial questionnaires assessing the perceived capacity to achieve physical activities related to daily and work activities, including material handling, postural tolerance, and ambulation [15]. The HFS (62 tasks; range, 0–248) was used for patients with upper limb injuries [32, 33] and the SFS (50 tasks; range, 0–200) for patients with spinal or lower limb injuries [34, 35]. To be able to compare the scores, we rescaled the HFS score to a maximum of 200 points. The higher the score, the higher the perceived functional ability.

### 2.5. Contributing Factors

#### 2.5.1. POAM-P

Avoidance, pacing, and overdoing/persistence were assessed at entry using POAM-P, developed by Cane [5] and validated in French [36]. The POAM-P has good psychometric properties and identifies in clinical practice the 3 main activity patterns, all of which demonstrated excellent internal consistency (Cronbach's alpha coefficients: 0.88, 0.89, and 0.85 for avoidance, pacing, and overdoing subscales, resp.) [36]. Directions are “People who have pain use different ways to do their daily activities. Think about how you usually do your daily activities.” Examples of item content include “I stop what I am doing when my pain starts to get worse” (avoidance), “I go back and forth between working and taking breaks when doing an activity” (pacing), and “When I am doing an activity I don't stop until it is finished” (overdoing). The instrument includes 30 questions (10 for each pattern), each scored from 0 to 4 points, and people have to determine the extent to which the proposal fits them. Each subscale is quoted individually. The highest rating reflects a favorite, but not exclusive, behavioral pattern (range: 0–40 points). Without a validated pattern classification methodology, each subscale is handled with continuous scores according to previous research [5, 22, 37, 38].

### 2.6. Outcomes

#### 2.6.1. FCE

The FCE helps determine the individual's safe functional capacity, a very important perspective for people with chronic pain who may be subject to fear of reinjury. Four of the FCE tests with high reliability were conducted during the rehabilitation program, according to Workwell–Isernhagen FCE standards [39, 40]: the floor-to-waist, the waist-to-overhead, the horizontal lifts, and the dominant-handed carrying test. FCE were performed by FCE certified physical therapists who had no knowledge of the POAM-P score and differed from the attending therapist to reduce the risk of bias. During the FCE load-carrying tests, the FCE assessor determines patient's maximum capacity after around five load increases of 2.5 or 5 kg for each increment. As already detailed [15], to validate a step, the load must be lifted five times for the floor-to-waist, the waist-to-overhead tests, or horizontal lift and carried over a distance of 15 m for the dominant-handed carrying test. Each step must be performed within 90 seconds, before the assessor increases the weight. Maximal safe performance is determined by the FCE assessor and is based on biomechanical and physiological signs in response to the effort: bulging of prime movers and accessory muscles, very wide base, counterbalance, increase in heart and respiration rate, very slow pace, and safe lifting but inability to maintain control with the addition of any more weight [41]. Unless maximal performance is reached, the test may be interrupted for safety reasons by the assessor or by the patients themselves if they feel unable to perform the task [15].

### 2.7. Data Collection

To minimize the measurement bias, questionnaires and clinical and demographic data were collected during the 2 days following admission, patients being planned for two 30-minuteperiods devoted to questionnaires. The use of a digital pen allowed data capture and transfer from paper to the data files. FCEs were performed 3 to 4 weeks after admission and were done under the supervision of certified physical therapists, familiar with the tests through regular training and clear instructions.

### 2.8. Statistical Analyses

Descriptive statistics were expressed as mean and standard deviation for continuous variables, whereas median and interquartile range were used if the distribution was skewed after visual inspection of data. Categorical variables were expressed as count and percentage.

We compared the POAM-P scores according to sociodemographic variables with analysis of variance or Student's *t*-test for binary variables. Eta-squared coefficient was expressed to determine and compare each categorical effect. Following Cohen [42], eta-squared effect size cutoffs were used: 0.01 for small, 0.06 for medium, 0.14 for large effect.

We looked for Pearson's or Spearman's correlations between the POAM-P scores and other variables, following the distribution of variables. Evans's [43] strength of correlations was used: less than 0.20 for very weak, 0.20 to 0.39 for weak, 0.40 to 0.59 for moderate, and 0.60 or greater for a strong correlation.

To study the association between activity patterns and the FCE tests, we used continuous scores of each of the three subscales of the POAM-P [5, 22, 37, 38]. The four FCE tests selected were not available for all patients. A total of 304 patients underwent the floor-to-waist lift, 303 performed the waist-to-overhead lift, 271 performed the horizontal lift, and 298 performed the dominant-handed carrying test. Standard multiple linear regression models were applied to measure the associations between FCE tests and activity patterns (POAM-P subscales) while adjusting these associations for potential confounders. Age, gender, BMI, pain severity, trauma severity, localization of injury, and education were chosen for their potential influence on outcome and activity patterns. The available sample size allowed the chosen parameters in the regression models to keep a minimum of 15 observations per parameter [44]. Only patients with complete data were included in the analysis. As patients did not have to perform every lifting test, the number of cases with complete data may differ for each analysis. To compare effects of variables on the outcome, standardized coefficients are presented [45]. The significance level was set as a probability less than 0.05. All analyses were performed using Stata 15.1 (StataCorp, College Station, Texas, USA).

## 3. Results

### 3.1. Sample Characteristics

A total of 304 patients were included. Patients were predominantly middle-aged men (mean age, 42.3 years; 94.7% men), nearly half were not French native speakers, and 42% had a low level of education. The median duration between trauma and rehabilitation was 15 months (interquartile range, 10–23 months). Injury locations were 44.2% upper limb, 42.2% lower limb, 11.5% spine injury, and multiple sites for the rest. Most injuries occurred at work (54.3%) and were classified as minor or moderate according to the Abbreviated Injury Score. The mean pain severity assessed by BPI was 4.03 ± 1.76, and the mean pain interference with daily activity was 4.1 ± 1.97. The HADS score was just below 7 ± 4.05 points for depressive symptoms and 9 ± 3.98 points for anxiety symptoms. When looking at perceived ability to perform working tasks assessed with the SFS/HFS, most patients felt they could perform light work.  Table 1 details patients' characteristics and their scores for the different psychological questionnaires as well as POAM-P scores for activity patterns. For the entire cohort, the mean maximal performances on the FCE tests were 22.11 ± 10.38 kg for the floor-to-waist lift, 13.74 ± 6.83 kg for the waist-to-overhead lift, 25.43 ± 10.38 kg for the horizontal lift, and 18.70 ± 8.21 kg for the dominant-handed carrying test.

Higher avoidance scores were found in nonnative French-speaking patients, whereas lower overdoing/persistence scores were found in patients with lower limb trauma. Effect sizes were small-to-medium (Eta-squared) [42] (Table 2).

Correlations between POAM-P subscales and other variables are presented in Table 3. The avoidance score was moderately correlated with pacing (*r* = 0.5), whereas only weak correlations were found for pacing and overdoing subscales with other variables (Table 3).

### 3.2. Relationship between Patterns and Observational Functional Outcomes

Results of the multivariable model analysis are presented in Table 4. The overdoing/persistence pattern was found to have a significant influence on the 4 FCE tests, after adjustment for confounding variables. For the floor-to-waist lift, a high overdoing/persistence score was associated with higher performances (coefficient = 0.20; *p*=0.001), meaning that for each supplementary point at the overdoing/persistence score (scale from 0 to 40 points), an increase of 0.20 kg is expected for the maximal lifted weight. In other words, a difference of 2 kg at the maximal lifted weight is expected between patients having a difference of 10 points on the overdoing/persistence score. When looking at the standardized coefficient, 3.28 kg represents the increase expected for the maximal floor-to-waist lift for a gain of 2 standard deviations (16.42 points) at the overdoing/persistence score. We found no influence of avoidance and pacing patterns.

For the waist-to-overhead lift, similar associations were found. A high overdoing/persistence score was associated with higher performances (coefficient = 0.13; *p*=0.004), meaning that for each supplementary point at the score, there will be an increase of 0.13 kg. When looking at the standardized coefficient, an increase of 2.08 kg is expected every 16.42 points. We found no influence of avoidance and pacing patterns.

For the horizontal lift, a higher overdoing/persistence score was also associated with higher performance (coefficient = 0.31; *p* < 0.001), meaning that for each supplementary point on the 40-point scale, an increase of 0.31 kg is expected. When looking at the standardized coefficient, an increase of 5.15 kg is expected every 16.42 points (2 SD) for the maximal horizontal lift.

For the dominant-handed carrying test, overdoing/persistence was again positively associated with performance (coefficient = 0.19; *p*=0.001) as opposed to pacing, which was negatively associated (coefficient = –0.14; *p*=0.034). A difference of 1 point on each questionnaire, respectively, represents an increase of 0.19 kg or a loss of 0.14 kg on the maximal carrying test. We found no influence of avoidance.

## 4. Discussion

Activity patterns have an influence on physical performance. As expected, our hypothesis regarding overdoing/persistence was verified, as it was positively associated with the 4 FCE tests. Pacing was found to be negatively associated only with carrying with the dominant-hand, but no association was found for the 3 other tests, whereas avoidance was not found to be associated with any of the 4 FCE tests.

Overdoing/persistence was associated with the 4 FCE tests studied. An increase of 10 points on the overdoing/persistence score represents an increase ranging from 1.3 kg for maximal waist-to-overhead lift to 3.1 kg for maximal horizontal lift. As weights have to be carried 5 times to validate each step for the lifting tests, the differences for the total manipulated weights are 5 times heavier: 6.5 kg for the maximal waist-to-overhead lift to 15 kg for the maximal horizontal lift for an increase of 10 points on the overdoing/persistence score. When looking at the standardized coefficient that allows comparison of numeric and binary variables, the expected gain for an increase of 16 points at the overdoing/persistence score (2 SD) ranged from 2.1 kg for the maximal waist-to-overhead lift to 5.1 kg for the maximal horizontal lift. These gains counterbalance the expected loss of performance associated with age, as the expected change for age ranged from a 1.9 kg loss for the carrying with dominant-hand test to 3.4 kg for the floor-to-waist lift, for every increase of 22 years (2 SD). If we compare the effect with gender, it also partially compensates for the influence of female gender on physical performances during FCE.

The influence of the overdoing/persistence pattern is debated in the literature, which is mainly based on subjective measures: Cane et al. found a positive association with disability in a population of patients with chronic pain recruited from a pain clinic, with a median pain duration of 7 years, and a high proportion of fibromyalgia, in contrast to our population [5], whereas others found mixed associations of task persistence [6, 8], or no association [47]. The association of overdoing/persistence with better functioning seems to be in accordance to those of Hasenbring, who found a lower perceived disability in a subgroup of patients called “eustress-endurant” [48], and with Luthi who found positive associations of overdoing/persistence with functioning [22]. Interestingly in a recent publication, Cane found that overdoing patients had a higher perceived functioning, assessed with the Pain Disability Index compared with patients with high avoidance and pacing subscales [3]. This difference with a previous study [5] is not discussed by Cane but may be linked with the fact that patients were grouped in clusters in the latter study, whereas an analysis of each subscale was done in the former. An association of overdoing/persistence patterns of activity with observational measures was found in few studies: Huijnen et al. could not find an association of patterns with daily life activity measured with accelerometers in patients with chronic low-back pain [12], whereas Andrews et al. found an association between perceived overactivity and physical activity measured with accelerometers [49]. These contradictory results may be explained by the different settings and populations of patients of the studies and the various strategies that may be used depending on the context [4, 50]. In the present study patients were asked to give their maximal safe effort. High demand instructions have been shown to improve reliability of tests [51] and even if instructions to patients may influence strength measurements [51–53], the specific influence of a demand on a preferential activity pattern is not known. We suppose high demand instructions improve performance of every patient and we do not think it would annihilate pacing or avoidance patterns, as we found similar associations in a former study addressing physical fitness tests without the demand to give a maximum effort [22].

We found no significant association of pacing with the 3 FCE lifting tests. Other studies using the POAM-P and addressing subjective measures of functioning showed contradictory results: Cane et al. found lower perceived disability for patients with a high level of pacing [5], whereas Kindermans found pacing to be positively correlated with disability [6]. The negative association with maximal carrying with dominant-hand performance may be in accordance with results found in another study in the same setting, in which high pacing was associated with lower walking performances [22]. This association with lower physical performance may be consistent with other studies, which found pacing to be associated with disability [4] or lower level of physical activity [54]. We have no definite explanation about the fact that pacing was found to be negatively associated with only one of the four tests. Our hypothesis is that behavioral strategies vary according to the context [4]. When looking at the 4 studied FCE tests, the dominant-hand carrying test may indeed represent a more complex activity compared to the other 3 tests. It requires not only lifting a weight from one point to another in incremental stages every 5 succeeded lifts but also walking for 15 m while carrying a weight that will increase with every succeeding stage. Moreover, the test itself takes longer than the 3 lifting tests, and pain-contingent pacing addressed with the POAM-P might become preferential in this situation, as opposed to other lifting tasks that may not promote it.

We found no association between the avoidance pattern and any of the 4 FCE tests. These results may seem surprising at first glance, as this pattern is associated with poor functioning in the literature [7, 15, 47, 55]. However, the few studies that also used observational measures of physical activity showed that there was more often no significant influence [10, 12, 22]. This discrepancy between subjective perception and observation can have a major influence/impact on the management of these patients. Indeed, successfully challenging these perceptions by comparing them with the actual performance achieved during a rehabilitation program, for instance, could be of great help in the care process. For some patients, this could lead to a more realistic interpretation of their own situation, especially among those who favor avoidance or pain-contingent pacing strategies [3, 22].

### 4.1. Implications of This Study

Our results highlight the importance of using observational measures in studies addressing the influence of behavioral patterns in patients with chronic pain. Such measures seem important, as subjective tools cannot just replace them and may lead to inaccurate or incomplete conclusions [56]. These observational measures are moreover not so costly and time-consuming and can be easily integrated into a therapeutic program often performed by physiotherapists or occupational therapists for their functional component [56].

There is a positive association of overdoing/persistence pattern with objective physical activity measurements. Even if we cannot draw causal interpretation, our results suggest a behavioral strategy based on task-contingent persistence instructions may be more effective than modulation strategies often proposed in the rehabilitation of chronic pain [7]. Without asking patients to exert themselves beyond their limits (excessive persistence), the positive associations of task-contingence persistence with physical performance should promote strategies that enable choosing an activity, precisely determining the level of effort at which it can be safely performed, and setting the training conditions that will allow the patient to complete this task, without pain or fatigue being grounds for stopping the activity. It would be interesting to evaluate whether this approach makes it possible to break the association between pain, fatigue, and disability that often remains observed, including therapeutic interventions based on activity modulation [57].

### 4.2. Strengths and Limitations

This is the first study addressing measures of activity patterns in the context of patients with chronic musculoskeletal pain after trauma. The use of FCE as an observational measure is another strength because of its reliability and ability to determine a maximum safe effort for the patient. However, this study also has some limitations. First, the sample mainly consisted of men addressed for specialized rehabilitation. The setting in itself may influence activity patterns and indeed may limit the generalization of our results. This type of study should therefore be repeated in samples that include a greater number of women. This would be all the more interesting as the overdoing/persistence pattern has been positively associated with the female gender [5]. A second limitation may be the interval between activity pattern evaluation with the POAM-P and FCE tests, as activity patterns may change during the rehabilitation program [3]. However, the associations found indicate that the activity patterns predict future behavior. Another limitation is related to the POAM-P questionnaire and the interpretation of overdoing/persistence. For Kindermans et al. [6], the POAM-P, as compared with other questionnaires that can be used, is thought to reflect task-contingent persistence more than excessive persistence or pain-contingent persistence, which may have a worse outcome. Finally, this observational study and the type of analysis conducted allows us to state an influence of some activity patterns on FCE performances but does not enable us to establish a cause-effect relationship.

## 5. Conclusion

Our results suggest that the overdoing/persistence pattern as defined in the POAM-P is positively associated with the following functional tasks: carrying a load from the ground to the waist, carrying a load from the waist to the head, lifting a load horizontally, and moving a load with the dominant hand, all of which are common tasks in daily and professional life. Further studies will be needed to determine whether there is a causal relationship between this pattern and physical performance.

## Figures and Tables

**Table 1:** Summary statistics.

Type of variable	Variable	*N*	Possible values	
Biological	Age	304	Years	42.3 (11.18)
	Gender	304	Female	16 (5.3%)
			Male	288 (94.7%)
	BMI	300	kg/m^2^	27.87 (4.7)
	Injuries location	303	Spine	35 (11.5%)
			Upper limb	134 (44.2%)
			Lower limb	128 (42.2%)
			Multiple	6 (2.0%)
	AIS (injury severity)	299	Minor	92 (30.8%)
			Moderate	169 (56.5%)
			Severe	38 (12.7%)
	Interval injury-rehabilitation	271	Days	559 (392)
	BPI severity subscale	303	0–10	4.0 (1.8)
	BPI interference subscale	303	0–10	4.1 (2.0)

Social	Native language	304	French	166 (54.6%)
			Other	138 (45.4%)
	Education level	303	Low	126 (41.6%)
			High	177 (58.4%)
	Employment contract	304	Yes	149 (49.0%)
			No	155 (51.0%)
	Work-related injury	300	Yes	163 (54.3%)
			No	137 (45.7%)

Anxiety/depressive symptoms	HADS-D	303	0–21	7.0 (4.0)
	HADS-A	302	0–21	8.8 (4.0)

Perceived disability	HFS/SFS	302	0–200	128.8 (38.6)

Predictors	Avoidance	304	0–40	28.8 (8.1)
	Pacing	304	0–40	26.1 (8.2)
	Overdoing	304	0–40	20.9 (8.2)

Outcomes	FCE floor-to-waist lift	304	0–50	22.1 (9.0)
	FCE waist-to-overhead lift	303	0–50	13.7 (6.8)
	FCE horizontal lift	271	0–60	25.4 (10.4)
	FCE carrying dominant-hand	298	0–50	18.7 (8.2)

*N* = available data for each variable; possible values = range for continuous variables and categories for dichotomized variables; descriptive statistics = mean value and standard deviation for continuous variables and absolute number and relative number for binary variables, age in years. BMI = body mass index (kg/m^2^); AIS = Abbreviated Injury Scale, interval between injury and hospitalization in days; BPI = Brief Pain Inventory; HADS-D = Hospital Anxiety and Depression Scale-depression subscale; HADS-A = Hospital Anxiety and Depression Scale-anxiety subscale; HFS = Hand Function Sort and SFS = Spinal Function Sort, normalized to 200 points; FCE = Functional Capacity Evaluation (kg).

**Table 2:** Mean POAM-P scores according to sociodemographic variables.

Variable	*N*	Possible values	Avoidance			Pacing			Overdoing		
			Mean	Eta-squared	*p* value	Mean	Eta-squared	*p* value	Mean	Eta-squared	*p* value
Gender	16	Female	27.5	0.0014	0.518	28.7	0.006	0.182	22.8	0.003	0.342
	288	Male	28.9			25.9			20.8		

Injuries location	134	Upper limb	28.6	0.014	0.244	26.0	0.018	0.143	21.1	0.028	0.037
	128	Lower limb	29.5			26.0			19.7		
	35	Back	27.5			27.7			23.6		
	6	Multiple sites	23.8			19.3			25.7		

AIS	92	Minor	29.6	0.010	0.220	26.2	0.000	0.955	20.5	0.002	0.786
	169	Moderate	29.1			26.0			21.0		
	38	Severe	26.9			26.4			21.5		

Native language	166	French	27.5	0.032	0.002	25.5	0.007	0.156	21.6	0.009	0.098
	138	Other	30.4			26.8			20.1		

Education	126	<9 years	29.2	0.002	0.455	26.9	0.007	0.149	20.5	0.001	0.528
	177	≥9 years	28.5			25.5			21.2		

Employment contract	149	Yes	28.8	<0.001	0.962	26.5	0.003	0.353	20.7	<0.001	0.688
	155	No	28.8			25.6			21.1		

Work-related injury	163	Yes	28.4	0.003	0.307	26.1	<0.001	0.890	20.3	0.007	0.157
	137	No	29.3			26.0			21.7		

Student's *t*-tests or analysis of variance to compare groups. POAM-P: Patterns of Activity Measures-pain; AIS = Abbreviated Injury Scale. Effect size (eta-squared) [42]: 0.01 = small; 0.06 = medium; 0.14 = large.

**Table 3:** Correlations between the three POAM-P subscales and other variables.

Variable	Avoidance		Pacing		Overdoing	
	Correlation	*p* value	Correlation	*p* value	Correlation	*p* value
Avoidance	1.00		0.50	<0.001	−0.29	<0.001
Pacing	0.50	<0.001	1.00		−0.12	0.033
Overdoing	−0.29	<0.001	−0.12	0.033	1.00	
BPI-interference	0.31	<0.001	0.05	0.427	−0.16	0.004
BPI-severity	0.28	<0.001	0.07	0.253	−0.17	0.002
HADS-D	0.25	<0.001	0.01	0.866	−0.14	0.012
HADS-A	0.13	0.052	−0.05	0.352	−0.05	0.402
HFS/SFS	−0.23	<0.001	−0.15	0.007	0.23	<0.001
Age	−0.01	0.851	0.27	<0.001	−0.07	0.253
FCE low lift	−0.11	0.121	−0.18	0.001	0.21	<0.001
FCE horizontal lift	−0.09	0.209	−0.21	<0.001	0.27	<0.001
FCE high lift	−0.06	0.384	−0.15	0.008	0.16	0.007
FCE carry dom. hand	−0.06	0.374	−0.17	0.004	0.22	<0.001

BPI = Brief Pain Inventory; HADS-D = Hospital Anxiety and Depression Scale-depression subscale; HADS-A = Hospital Anxiety and Depression Scale-anxiety subscale; HFS = Hand Function Sort and SFS = Spinal Function Sort, normalized to 200 points; FCE = Functional Capacity Evaluation (kg). Correlation coefficient [46]: 0.20 to 0.39 = weak; 0.4 to 0.59 = moderate; 0.60 and greater = strong.

**Table 4:** Multiple regression models for FCE performances.

Outcome	Variable	*n*	Adj. *r*^2^	Coeff.	CI (95%)	*p*	Std. coeff.
FCE floor-to-waist lift	Avoidance	293	0.25	−0.00	−0.14; 0.14	0.984	−0.02
	Pacing			−0.07	−0.21; 0.06	0.278	−1.22
	Overdoing			0.20	0.08; 0.32	0.001	3.28

FCE waist-to-overhead lift	Avoidance	292	0.31	0.06	−0.04; 0.16	0.211	1.03
	Pacing			−0.07	−0.16; 0.03	0.184	−1.09
	Overdoing			0.13	0.04; 0.21	0.004	2.08

FCE horizontal lift	Avoidance	260	0.24	0.08	−0.09; 0.26	0.358	1.33
	Pacing			−0.15	−0.31; 0.02	0.083	−2.43
	Overdoing			0.31	0.16; 0.47	<.001	5.15

FCE carrying dominant-hand	Avoidance	288	0.16	0.09	−0.04; 0.22	0.173	1.49
	Pacing			−0.14	−0.27; −0.01	0.034	−2.32
	Overdoing			0.19	0.07; 0.30	0.001	3.11

Adjusted with confounding variables: age, body mass index (BMI), gender, trauma location, trauma severity, pain, and education. Std. coeff.: standardized coefficient for a change of 2 SD.

## Data Availability

The data used to support the findings of this study are available from the corresponding author upon request.
